# Effectiveness of animation as a learning tool in applied medical sciences education: A comparative cross-sectional study among university students

**DOI:** 10.12669/pjms.41.4.11204

**Published:** 2025-04

**Authors:** Faisal Mousa Alzahrani

**Affiliations:** Dr. Faisal Mousa Alzahrani, PhD. Department of Clinical Laboratory Sciences, College of Applied Medical Sciences, Imam Abdulrahman Bin Faisal University, Dammam, Saudi Arabia

**Keywords:** Animation, Learning tool, Medical education, Saudi Arabia, University students

## Abstract

**Background & Objectives:**

This study investigates the effectiveness of animation compared to traditional lecture methods on student performance, cognitive outcomes, and knowledge retention among Applied Medical Sciences University students.

**Methods:**

In this cross-sectional comparative study, a total of 110 students from the College of Applied Medical Sciences (CAMS), Imam Abdulrahman bin Faisal University participated in the study conducted between 1^st^ April to 30^th^ June 2024. Participants were divided into two groups: one receiving traditional lecture-based instruction and the other receiving animated instructional content. Quizzes were structured into two components to evaluate knowledge retention and cognitive skills. The study further analyzed performance, gender differences, and academic year to assess the efficacy of animation.

**Results:**

The animation group outperformed the traditional group in overall scores of 72.48±10.13 vs. 61.16±12.30, (p=0.001), and the passing rate scaled were 60% and 80%. Largely significant differences were seen in male students (p=0.002) and fourth-year students (p=0.001) in the animation group. Knowledge and cognitive assessments showed statistically significant gains in the animation group compared to traditional methods (p< 0.05).

**Conclusion:**

Animation-based learning significantly enhances student performance, particularly in cognitive tasks, and is especially beneficial for fourth-year students. This highlights the effectiveness of animation as a teaching tool. Incorporating it can enhance learning outcomes and academic success in applied medical sciences education.

## INTRODUCTION

Animation has increasingly become a prominent academic tool in recent years across diverse academic disciplines, particularly in the applied health sciences. Historically, the foundation of teaching in these fields has relied on techniques such as lectures and static visuals. The growing complexity of medical information and the necessity for improved student engagement and understanding have encouraged educators to investigate alternate teaching methodologies. Animated multimedia, which conveys information dynamically and interactively, has demonstrated ability to enhance students’ cognitive abilities and knowledge retention.^1^-^3^

Research suggests that animations enhance students’ comprehension of intricate subjects and elevate their motivation to learn.^4^,^5^ In medical education, where comprehension of abstract and intricate processes is essential, multimedia learning aids like animations have become indispensable for picturing complex biological processes that conventional teaching methods frequently fail to depict adequately.^6^,^7^ For example, animations might depict the step-by-step mechanism of viral replication, the real-time flow of blood through cardiac chambers, or the spatial relationships between organs during surgical procedures. These tools differ fundamentally from basic PowerPoint slide transitions or simple GIFs, as they integrate motion, sound, and sequential storytelling to model dynamic systems. Research has shown that animations promote comprehension, engagement, and learning outcomes more effectively than static visuals or lectures.^8^

Cognitive theories of multimedia learning advocate for the utilization of animation, positing that animated content alleviates cognitive load by delivering information in a digestible and visually engaging manner.^9^ These ideas emphasize how animations improve visual and spatial comprehension, resulting in enhanced retention and application of knowledge. This is particularly pertinent in higher education, as students are frequently required to rapidly comprehend complex concepts, especially in health sciences courses. Moreover, animations accommodate many learning methods, rendering them an inclusive resource for heterogeneous classrooms.

Although the advantages of animation are well-documented, there is a small number of study examining how their efficacy differs across various academic levels, especially for cognitive and knowledge domains. Comprehending the influence of animations on students at different educational levels is essential for enhancing teaching approaches. The extent to which students in early academic years benefit comparably to their senior colleagues remains ambiguous, as does the effectiveness of animation in enhancing higher-order cognitive skills, such as critical thinking and analysis, relative to its impact on basic knowledge retention and can animations support higher-order cognitive skills (e.g., diagnostic reasoning) as effectively as they reinforce factual recall?

This study investigates these unresolved questions by comparing animation-based instruction to traditional lecture methods across different year levels in a health sciences program. The research problem centers on whether animations disproportionately enhance learning outcomes for specific academic cohorts or cognitive domains. Moreover, comprehending the function of animation may guide curriculum development and assist educators in customizing instructional methodologies to enhance the efficacy of animation for students at different educational levels.

## METHODS

This study employed a cross-sectional comparative study design to evaluate the difference between animation-based and traditional teaching methods on student’s academic performance across different levels. A total of 110 students from the College of Applied Medical Sciences (CAMS), Imam Abdulrahman bin Faisal University participated in the study conducted between 1^st^ April to 30^th^ June, 2024. The participants included 44 (40%) second-year, 38(34%) third-year students and 28 (26%) fourth-year students (Table-I).

**Table-I:** The demographics of the study participants.

Demographics		CAMS	
		Traditional n (%)	Animation n (%)
Gender	Male	25 (47)	26 (46)
	Female	28 (53)	31 (54)
Year	2^nd^	22 (42)	22 (39)
	3^rd^	19 (36)	19 (33)
	4^th^	14 (26)	14 (25)

### Ethical considerations:

The Imam Abdulrahman bin Faisal University institutional review board granted ethical clearance IRB-2024-3-265 dated March 27, 2024. Before starting the study, all participants provided their informed consent. The Declaration of Helsinki was followed in the conduct of the study, ensuring the confidentiality and anonymity of participants throughout the research process.

### Participants:

All participants were recruited from CAMS of the university. Among the participants, 51 (46%) were male and 59 (54%) were female. All students registered in that academic year were included in the current study. students were divided randomly into two groups of 55 each. Group-I presented with the traditional teaching method whereas Group-II was offered an animated-based teaching method.

### Intervention:

The intervention consisted of the implementation of visual aids, specifically animations, during instructional sessions. The assessments focused on cognitive skills and knowledge retention, measured through written quizzes administered immediately following both instructional sessions.

### Data collection:

Performance data were collected through written quizzes. The total time taken by students to complete the quiz was 10 minutes. Scores were analyzed and presented in various formats, including average scores, percentage distributions, and relative grades. The performance of students was further classified based on grade categories: A (90-100), B (80-89), C (70-79), D (60-69), and F (0-59).

### Evaluation of cognitive and knowledge performance:

The participant’s performances were further assessed in two specific domains: cognitive skills and knowledge. The scores for these domains were analyzed separately across the same year levels to determine the difference between both instructional methods.

### Statistical analysis:

Data was entered in an Excel sheet from the exam papers and then transferred to SPSS (statistical package for social science) version 24, IBM USA, for analysis. Average scores with standard deviations, frequency, and percentages were calculated. The normality of data was checked using Shaprio Wilk’s Test and *p* values less than 0.05 indicated that data is significantly deviating from the normal distribution. Therefore, a non-parametric student t-test (Mann Whitney test) was applied to investigate the performance differences between the groups and Chi-Square was used for categorical variable comparison. A *p*-value of less than 0.05 was considered statistically significant.

## RESULTS

A total of 110 students participated in the study, and the demographic information of all participants is displayed in Table-I. Group-I has 53 students who received traditional teaching methods, and Group-II consists of 57 participant who were taught using animation. The proportion of female students composed the majority in both categories (53% and 54%, respectively).

### Impact of Animation on Student Performance:

The comparison of student performance between traditional and animation across different demographic groups is presented in Table-II. Male students showed a statistically significant increase in performance with animation (72.64+8.24) compared to those traditional methods (62.82+12.09) (p=0.002). While female students also exhibited a slightly higher score (70.91+9.9) compared to their performance without animation (69.81+12.06), the difference was not statistically significant (*p*=0.384).

**Table-II:** Performance of students with and without animation.

Demographics		Traditional Mean+SD	Animation Mean+SD	p-value
Gender	Male	62.82+12.09	72.64+8.24	0.002*
	Female	69.81+12.06	70.91+9.9	0.384
Year Level	2^nd^	60.15+12.52	73.45+10.31	0.001*
	3^rd^	61.10+12.34	71.23+11.24	0.001*
	4^th^	62.82+12.09	72.64+8.24	0.004*
Overall		61.16+12.30	72.48+10.13	0.001*

* Statistically significant at 0.05.

At the academic level, significant differences were observed across all year levels. Second-year students demonstrated a surge in performance from a mean score of (60.15+12.52) traditional to (73.45+10.31) with animation (*p*=0.001). Similarly, third-year students scored higher from a mean of (61.10+12.34) in traditional compared to the animation (71.23+11.24) (*p*=0.001), and fourth-year student’s performance was similarly higher from traditional (62.82+12.09) to (72.64+8.24) with animation (*p*=0.004). Overall, students who were shown animation performed significantly better (72.48+10.13) compared to those without animation (61.16±12.30), with an overall *p*-value of 0.001.

### Grade Distribution among Traditional and Animation Group:

The distribution of grades between traditional and animation groups is presented in Table-III. A statistically significant difference in the grade distribution was in both groups. The percentage of students achieving an A grade (90-100) differ from 0% without animation to 4% with animation (*p*=0.022). The number of students were significantly higher, who achieved more than 70% in animation group compared to traditional group (36%).

**Table-III:** Distribution of grades among traditional and animation groups.

Students Grade	Traditional	Animation	p-value
A (90-100)	0(0)	4(4)	0.022*
B (80-89)	4(4)	22(20)	
C (70-79)	35(32)	40(36)	
D (60-69)	26(24)	22(20)	
F (0-59)	44(40)	22(20)	

* Statistically significant at 0.05.

### Cognitive and knowledge-based performance:

The effect of animation on cognitive and knowledge-based performance is summarized in Table-IV. Significant differences were observed in both cognitive and knowledge domains across all year levels. Cognitive performance in second-year students exhibits a higher cognitive score (71.66+10.4) without animation to (78.67+11.95) with animation, although this difference was not statistically significant (*p*=0.09). However, third-year students demonstrated slightly higher scores in cognitive domain, from (79.65+20.18) to (81.30+14.9), (p-0.06). On the other hand, fourth-year students showed a significantly higher performance, (67.35+14.6) without animation (86.76+10.36) with animation (*p*=0.001). Knowledge performance in the animation group across all year levels, significant differences were noted. Second-year students in Group-II showed higher performance (54.20+18.16) vs (74.5+15.3) respectively (*p*=0.001), third-year students (56.69+8.50) to (75.69+16.51) (*p*=0.023), and fourth-year students scored higher from (60.29+10.37) to (82.61+16.13) (*p*=0.001).

**Table-IV:** Effect of animation on knowledge and Cognitive.

Year	Cognitive		p-values	Knowledge		p-values
	Traditional Mean+SD	Animation Mean+SD		Traditional Mean+SD	Animation Mean+SD	
2^nd^	71.66+10.4	78.67+11.95	0.09	54.20+18.16	74.5+15.3	0.001*
3^rd^	79.65+20.18	81.30+14.9	0.06	56.69+8.50	75.69+16.51	0.023*
4^th^	67.35+14.6	86.76+10.36	0.001*	60.29+10.37	82.61+16.13	0.001*
Overall	73.63+16.7	85.0+13.18	0.001*	57.12+12.50	77.51+16.3	0.001*

* Statistically significant at 0.05.

### Grade Distribution Based on Cognitive and Knowledge Scores:

The distribution of grades based on cognitive and knowledge scores is detailed in Table-V. Statistically significant differences were observed in both domains following the introduction of animation. In terms of cognitive performance, the proportion of students earning an A grade was more in the animation group compared to traditional, 0% without animation to 24% with animation (*p*=0.001). Similarly, the percentage of students receiving F grades was very less in animation group (20% Vs 68%). For knowledge performance, 49% of students achieved an A grade in animation, compared to only 16% without animation (*p*=0.001). Furthermore, no students received an F grade with animation, compared to 16% who failed without animation.

**Table-V:** Distribution of grades obtained based on knowledge and cognitive.

Students Grade	Cognitive		p-values	Knowledge		p-values
	Traditional	Animation		Traditional	Animation	
A	0(0)	26(24)	0.001*	17(16)	54(49)	0.001*
B	2(1)	42(40)		49(45)	40(37)	
C	35(31)	15(13)		7(6)	8(7)	
D	0(0)	3(3)		19(17)	8(7)	
F	75(68)	22(20)		18(16)	0(0)	

* Statistically significant at 0.05.

**Fig.1:**
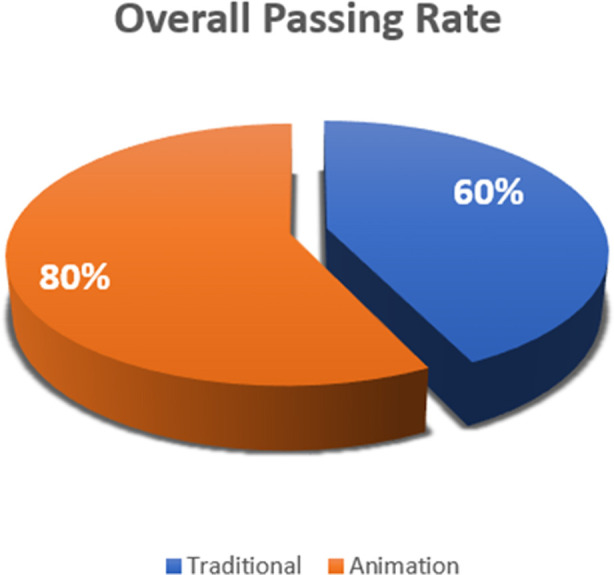
Rate of passing with and without animation.

The overall passing rate was significantly higher in animation groups as illustrated in Fig.1. This further supports the efficacy of animation in enhancing student learning outcomes.

## DISCUSSION

This study highlights the significant impact of animation-based instruction on student performance, particularly in applied medical sciences. The findings demonstrated significant disparities in academic performance, cognitive abilities, and knowledge retention among students exposed to animated content, corroborating prior research that suggests animations enhance learning efficacy.^10^

Nonetheless, certain studies indicate that animations are not invariably more successful than static images, particularly when static diagrams facilitate learning by enabling students to concentrate on specific features without the distractions that animations may present.^11^,^12^ This study observed gender-specific differences, with male students showing significant higher scores with animations (p=0.002). This corresponds with research indicating that gender serves as a crucial moderator: samples with fewer females and a higher proportion of males exhibited a stronger advantage from dynamic visuals.^13^ Nonetheless, it diverges from the findings of Mayer RE et al. and Moreno R et al.^7^, which indicate that all learners derive equal advantages from multimedia learning when cognitive burden is alleviated. This variation may be attributable to cognitive style, past technological exposure, or individual learning preferences.

The present study illustrates substantial performance enhancements across all academic tiers, with second-year students exhibiting the most pronounced cognitive advancements (p=0.001). This aligns with the findings of Bilginer EB et al. & Uzun E et al.^14^ and Mnguni L et al. & Moyo D et al.^2^, who illustrated the advantages of animations for early-year pupils facing challenges with abstract concepts. Nevertheless, alternative research indicates that as students’ progress, their dependence on visual assistance lessens, and the effectiveness of animations declines.^4^,^6^ This prompts an inquiry about whether animation should be employed selectively, prioritizing early-year pupils or those encountering difficulties with specific subjects. Students in the animation group exhibited elevated proportions of A and B grades (24% compared to 0% for A grades, p = 0.001) and a diminished failure rate (20% versus 68%, p = 0.001), reflecting patterns identified by Puspaningtyas ND et al. & Ulfa M et al.^15^ in integrated learning. The results underscore the motivational advantages of animations; yet many studies caution against potential cognitive overload in advanced learners if animations are not meticulously developed.^10^,^12^,^16^ This signifies that the design of animations must be meticulously aligned with the student’s cognitive abilities and the intricacy of the content to prevent adverse impacts on learning.

### Limitations:

Studying possesses certain limits. The study was first done in a single branch of the college, perhaps restricting the generalizability of the results. Subsequent research should encompass a broader and more heterogeneous sample across several universities. The assessment concentrated on specific elements of knowledge and cognitive domains; broadening the array of assessment instruments could yield a more thorough evaluation of learning outcomes. A delayed post-test was not administered to assess long-term information retention. Subsequent research ought to incorporate follow-up evaluations to gain a deeper comprehension of the enduring effects of animation-based pedagogy. Finally, the study failed to consider individual learning preferences, which may affect students’ responses to various teaching techniques.

## CONCLUSION

This study demonstrates the significant benefits of animation-based instruction in applied medical sciences, particularly for enhancing cognitive and knowledge domains. The effectiveness of animations varies across gender and academic levels, highlighting the need for a more nuanced, selective approach to instructional design. Further research with diverse samples is necessary to refine the application of animations and ensure their maximal benefit in education.
